# Senescence-related genes and proteins in the development of Alzheimer’s disease: evidence from transcriptomic and Mendelian randomization analysis

**DOI:** 10.3389/fnagi.2024.1423725

**Published:** 2024-08-02

**Authors:** Ying Liu, Jiao Chen

**Affiliations:** Department of Gerontology and Geriatrics, Shengjing Hospital of China Medical University, Shenyang, China

**Keywords:** Alzheimer’s disease, senescence, transcriptomic analysis, plasma proteome, Mendelian randomization

## Abstract

**Purpose:**

Alzheimer’s disease (AD) is a common neurodegenerative disease, which can lead to cognitive impairment and dementia. Since AD is tightly associated with aging and cellular senescence, objective of this study was to investigate the association between senescence-related genes and proteins (SRGs and SRPs) and the development of AD.

**Design:**

The whole study was based on transcriptomic analysis of control and AD brain tissues and Mendelian randomization (MR) analysis.

**Methods:**

For transcriptomic analysis, GSE5281 dataset from GEO database contains the transcriptomic data of human brain tissues (*n* = 161) from control group and AD patients. The expression of SRGs in control and AD brain tissues were compared by Student’s t test. For MR analysis, the instrumental single-nucleotide polymorphisms (SNPs) associated with 110 SRPs were filtered and selected from a large genome-wide association study (GWAS) for plasma proteome. The causality between plasma levels of SRPs and AD was explored using GWAS data of AD from Lambert et al. (17,008 cases and 37,154 controls) and further validated by using data from FinnGen consortium (6,489 patients and 170,489 controls). MR estimate was performed using the inverse-variance weighted (IVW) method and the heterogeneity and pleiotropy of results were tested.

**Results:**

Transcriptomic analysis identified 36 up-regulated (including *PLAUR*) and 8 down-regulated SRGs in AD brain tissues. In addition, the MR results at both discovery and validation stages supported the causality between plasma levels of PLAUR (IVW-*p* = 3.04E-2, odds ratio [OR] = 1.15), CD55 (IVW-*p* = 1.56E-3, OR = 0.86), and SERPINE2 (IVW-*p* = 2.74E-2, OR = 0.91) and the risk of AD.

**Conclusion:**

Our findings identified that *PLAUR*, as an SRG, may take part in the development of AD and found that high plasma levels of PLAUR was associated with increased risk of AD, indicating that this gene was a risk factor for this disease and providing the rationale of existing drugs or new preventative and therapeutic strategies.

## 1 Introduction

Worldwide, dementia has become a major public concern, whose prevalence is expected to reach 152 million by 2050 (Livingston et al., 2020), placing considerable health burden on affected individuals and economic burden on society. Notably, a significant proportion of dementia cases was caused in whole or in part by Alzheimer’s disease (AD) (Brookmeyer et al., 2011; Saez-Atienzar and Masliah, 2020). AD is a progressive neurodegenerative disease (NDD) leading to cognitive impairment, neuropsychiatric symptoms, disability, and even premature death (Demakis, 2007; Gillespie et al., 2013). Despite recent advances, the preventive or treatment options for AD remain limited.

Advancing age is a major risk factor of AD (Holloway et al., 2023). Cellular senescence is a hallmark of aging and a significant contributor to age-related diseases including AD (Holloway et al., 2023). The accumulation of senescent neurons and glial cells have been detected in the brains of AD patients and mouse models and that selective clearance of senescent cells has been reported to prevent tau pathology and improves cognition in AD mouse models (Bussian et al., 2018). However, the mechanisms underlying when and how cellular senescence contributes to AD pathogenesis remain unclear.

Importantly, a gene set of senescence-related genes (SRGs) was reported for predicting senescence-associated pathways and senescent status across tissues (Saul et al., 2022), used in many studies about age-related diseases (Doolittle et al., 2023; Farr, 2023; Yu et al., 2023; Lei et al., 2024). This list of SRGs contains 9 categories, including Metalloproteases, Cytokine/Chemokine, Growth factor, Intercellular signal molecule, Miscellaneous, Protease inhibitors, Protein modifying enzymes, Transcription factors and regulators, and Transmembrane signal receptors (Saul et al., 2022). Saul et al. (2022) also illustrated a dense interaction network encoded by there SRGs, where every gene was highly influenced by other members in this gene set in multiple styles such as ligand-receptor interactions (such as *EGF* and *EGFR*) and influence expression or secretion patterns (such as *TNFA* and *IL6*). This network of SRGs was considered to facilitate better characterization of senescent cells. Especially, Lei et al. (2024) used this gene set and identified potential biomarkers and drug targets for age-related macular degeneration (AMD), an NDD in the eye. However, the association between SRGs and the proteins encoded by them (senescence-related proteins, SRPs) and the development of AD has not been investigated and remains unclear.

**Figure 1:**
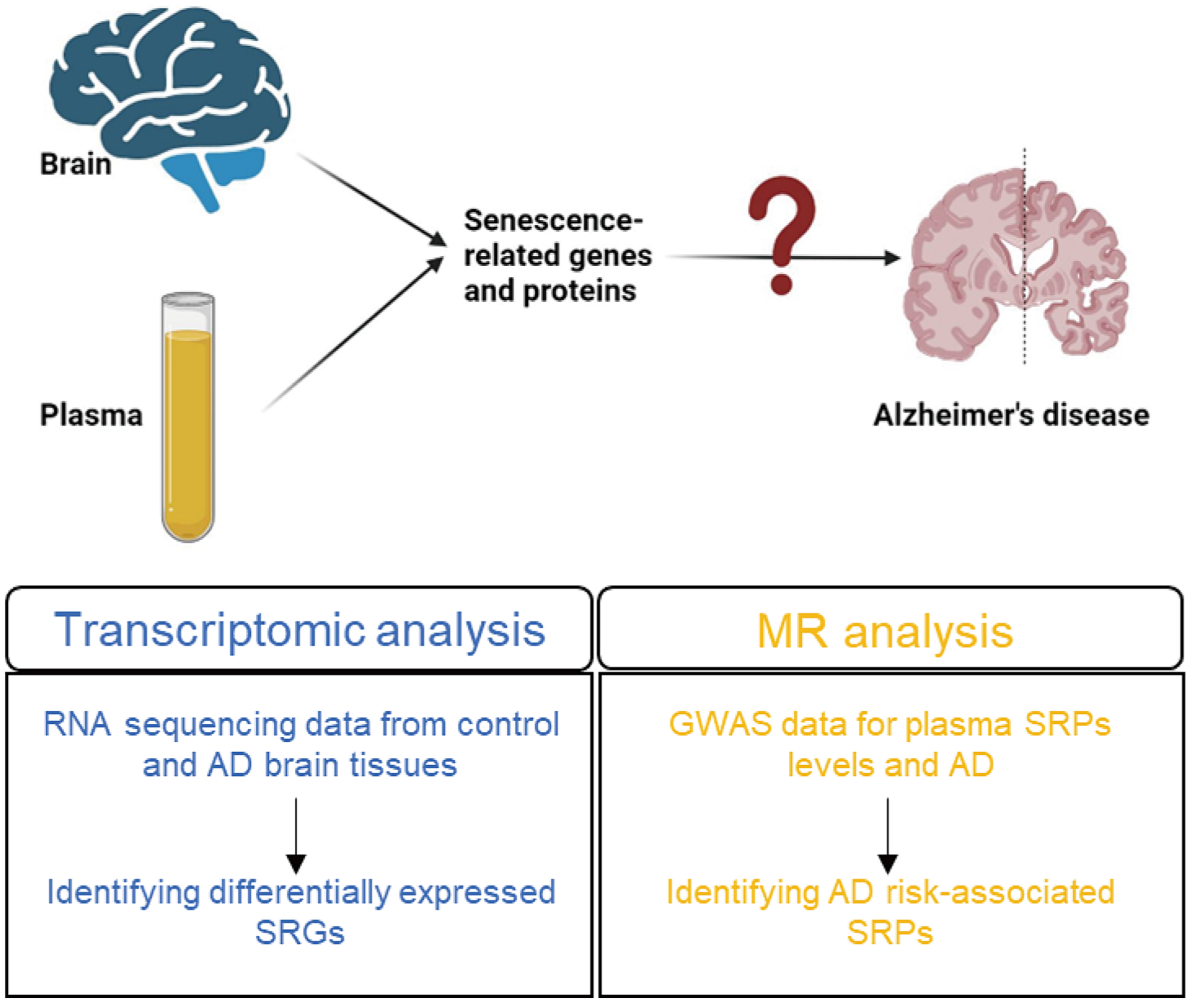
The summary of the study design. AD, Alzheimer’s disease; SRGs, senescence-related genes; MR, Mendelian randomization; GWAS, genome-wide association studies; SRPs, senescence-related proteins.

In this study, by combing transcriptomic and Mendelian randomization (MR) analysis, we explored the expression of SRGs in control and AD brain tissues and the causality between the plasma levels of SRPs and the risk of AD (Figure 1). Mendelian randomization (MR) approaches have provided the possibility and opportunities for researchers to identify risk or protective factors for various diseases (Davey Smith and Hemani, 2014), by using genome-wide association studies (GWAS) data and genetic variants as instrument. Moreover, MR approaches have been used to explore risk factors of AD (Li et al., 2023; Ning et al., 2023; Zhu et al., 2023). As a results, the expression of *PLAUR* was up-regulated in AD brain and the plasma level of protein encoded by *PLAUR* gene were identified to be associated with increased risk of AD.

## 2 Materials and methods

### 2.1 Transcriptomic analysis

The RNA sequencing data of human brain tissues were obtained from GEO database. GSE5281 reported the transcriptomic atlas of human brain tissues (*n* = 161) from control group and AD patients (Newman et al., 2012). The list of SRGs was shown in Supplementary Table 1 (Saul et al., 2022). The expression of SRGs in control and AD brain tissues were compared by student t test via limma R package. The criteria for differentially expressed genes (DEGs) were adjusted *p* < 0.05 and |log (FC) | > 1.

### 2.2 GWAS data acquisition for plasma proteins and AD

In this study, we used a two-sample MR approach (inverse-variance weighted, IVW) to explore relationship between SRPs plasma levels and risk of AD. The summary-level GWAS data of SRPs were obtained from Ferkingstad et al. (2021), a large-scale GWAS project on plasma proteome involving 35,559 participants. Summary-level GWAS data of AD were obtained from a meta-analysis of previously published GWAS datasets and reported by Lambert et al. (2013), which includes 17,008 AD cases and 37,154 cognitively normal age-matched controls. GWAS data of AD from FinnGen consortium (6,489 cases and 170,489 controls) (Kurki et al., 2003) was used for replication and validation.

### 2.3 Selection of genetic instruments

**Figure 2:**
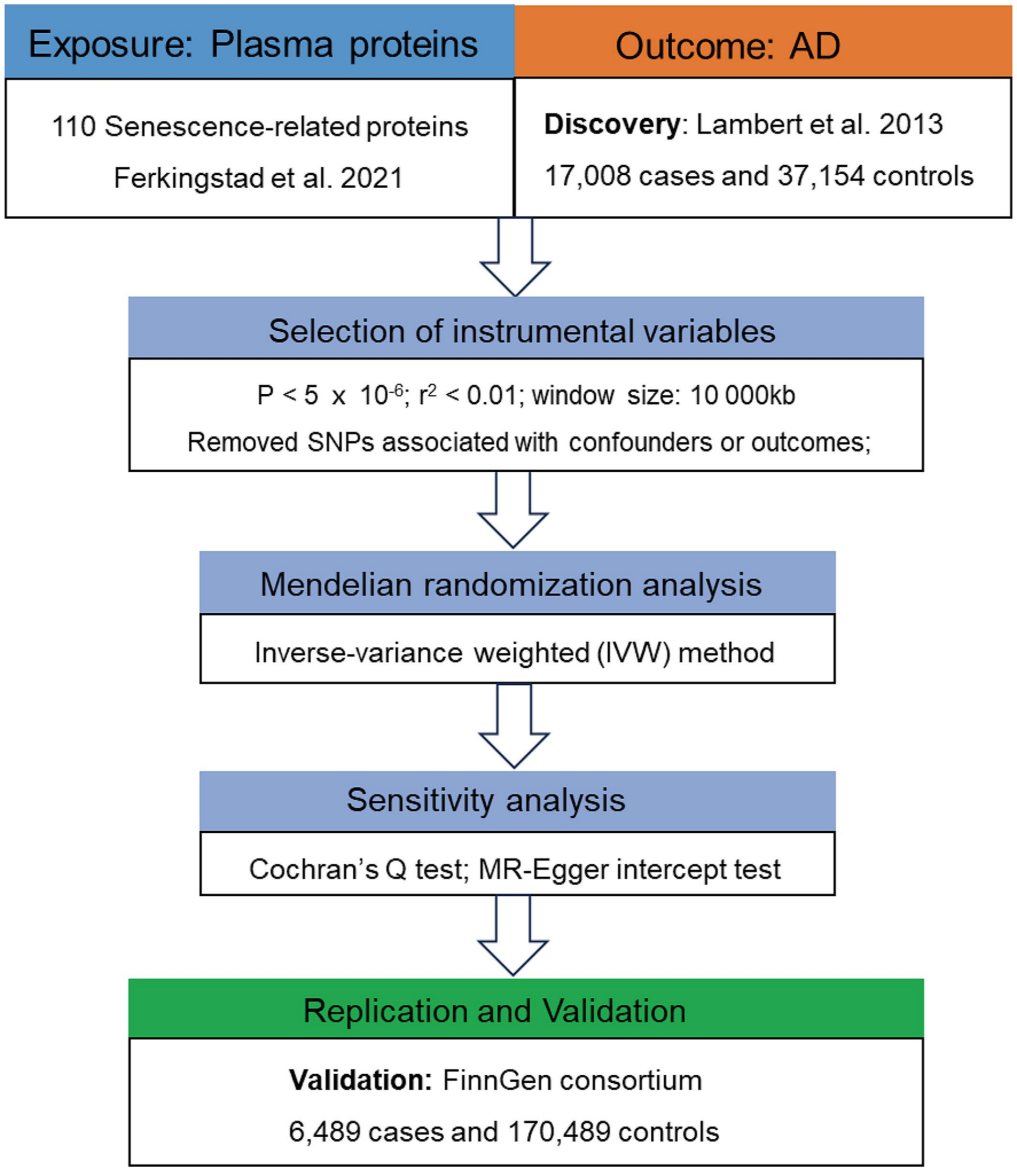
Flowchart of the MR analyses. MR, Mendelian randomization; SRPs, senescence-related proteins; AD, Alzheimer’s disease; SNP, single nucleotide polymorphism; IVW, inverse-variance weighted.

The flowchart of this study is presented in Figure 2. Following the acquisition of necessary GWAS data, we need to selected suitable genetic instrument for subsequent analysis. Genetic instrument for MR analysis, i.e., single-nucleotide polymorphisms (SNPs), were selected through the following process and criteria: (1) significantly associated with the exposures (*p* < 5 × 10^−6^); (2) linkage disequilibrium (LD) threshold (*r*^2^ < 0.01 and window size 10,000 kb); (3) F-statistic > 10.

### 2.4 Causality estimated by MR analysis

The control of pleiotropy is important for the validity of MR results (Lawlor et al., 2008). Therefore, we used the random-effect IVW method (Burgess et al., 2016) as the method for MR estimates and performed Cochran’s Q test and MR-Egger intercept test to evaluate the heterogeneity and detect pleiotropy (Bowden et al., 2019). Moreover, the MR Pleiotropy RESidual Sum and Outlier (MR-PRESSO) (Verbanck et al., 2018) test was used to further assess the robustness of MR estimations and SNPs identified as outliers by MR-PRESSO were removed. The cutoff used in these three tests was set as 0.05, where *p* > 0.05 means there was no apparent heterogeneity or pleiotropy.

### 2.5 Potential drugs identification

Potential drugs targeting the identified AD-associated plasma proteins were obtained from the Drug-Gene Interaction Database (DGIdb 5.0)^1^ (Cannon et al., 2024), which can output a list of potential drugs after you input a name of gene.

### 2.6 Statistical analysis

All analyses were performed in R (version 4.0.1) using the limma and TwoSampleMR (Hemani et al., 2018) R package. All statistical tests are two-sided. The cutoff for heterogeneity and pleiotropy tests was set as 0.05, where *p* > 0.05 means there was no apparent heterogeneity or pleiotropy. The code for MR analysis is accessible at https://mrcieu.github.io/TwoSampleMR/articles/index.html.

## 3 Results

### 3.1 Results of transcriptomic analysis

**Figure 3:**
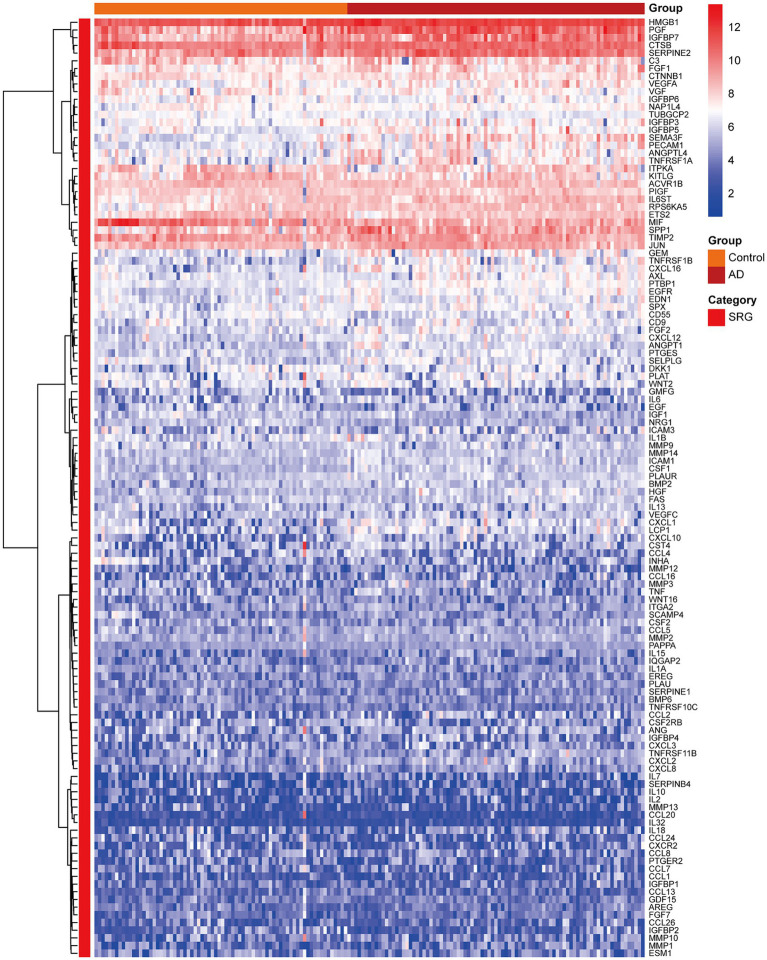
The heatmap showing the expression profiles of SRGs in control and AD brain tissues. SRGs, senescence-related genes; AD, Alzheimer’s disease.

**Figure 4:**
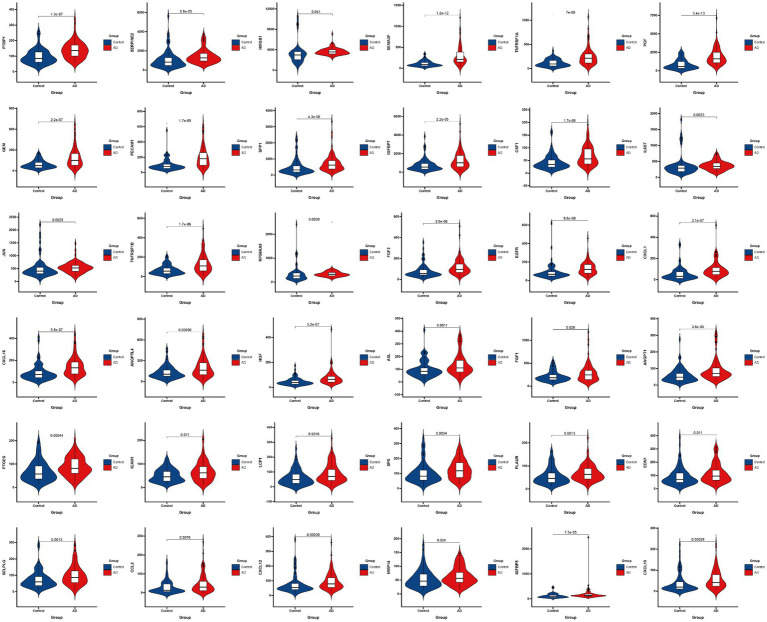
The violin plot of the 36 up-regulated SRGs in AD brain tissue. SRGs, senescence-related genes; AD, Alzheimer’s disease.

**Figure 5:**
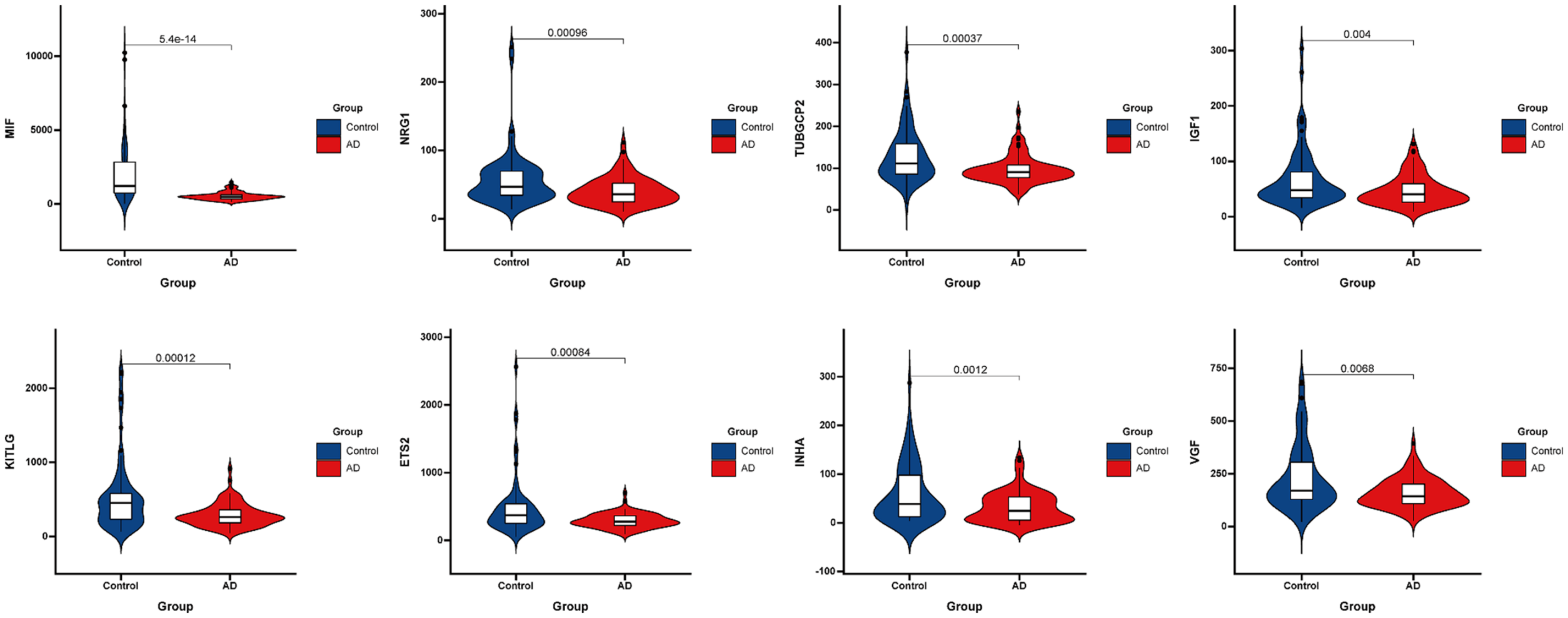
The violin plot of the 8 down-regulated SRGs in AD brain tissue. SRGs, senescence-related genes; AD, Alzheimer’s disease.

Figure 3 presented the heatmap of SRGs expression in control and AD brain tissues, showing that many SRGs were differentially expressed in these two groups. The 36 up-regulated SRGs in AD brain and the violin plot for them was shown in Figure 4, including *PTBP1, SERPINE2, HMGB1, SEMA3F, TNFRSF1A, PGF, GEM, PECAM1, SPP1, IGFBP7, CSF1, IL6ST, JUN, TNFRSF1B, RPS6KA5, FGF2, EGFR, CXCL1, CXCL16, ANGPTL4, HGF, AXL, FGF1, ANGPT1, PTGES, ICAM1, LCP1, SPX, PLAUR, EDN1, SELPLG, CCL2, CXCL12, MMP14, IGFBP5*, and *CXCL10*. The 8 down-regulated SRGs in AD brain and the violin plot for them was shown in Figure 5, including *MIF, NRG1, TUBGCP2, IGF1, KITLG, ETS2, INHA*, and *VGF*.

### 3.2 Discovery stage MR results

**Figure 6:**
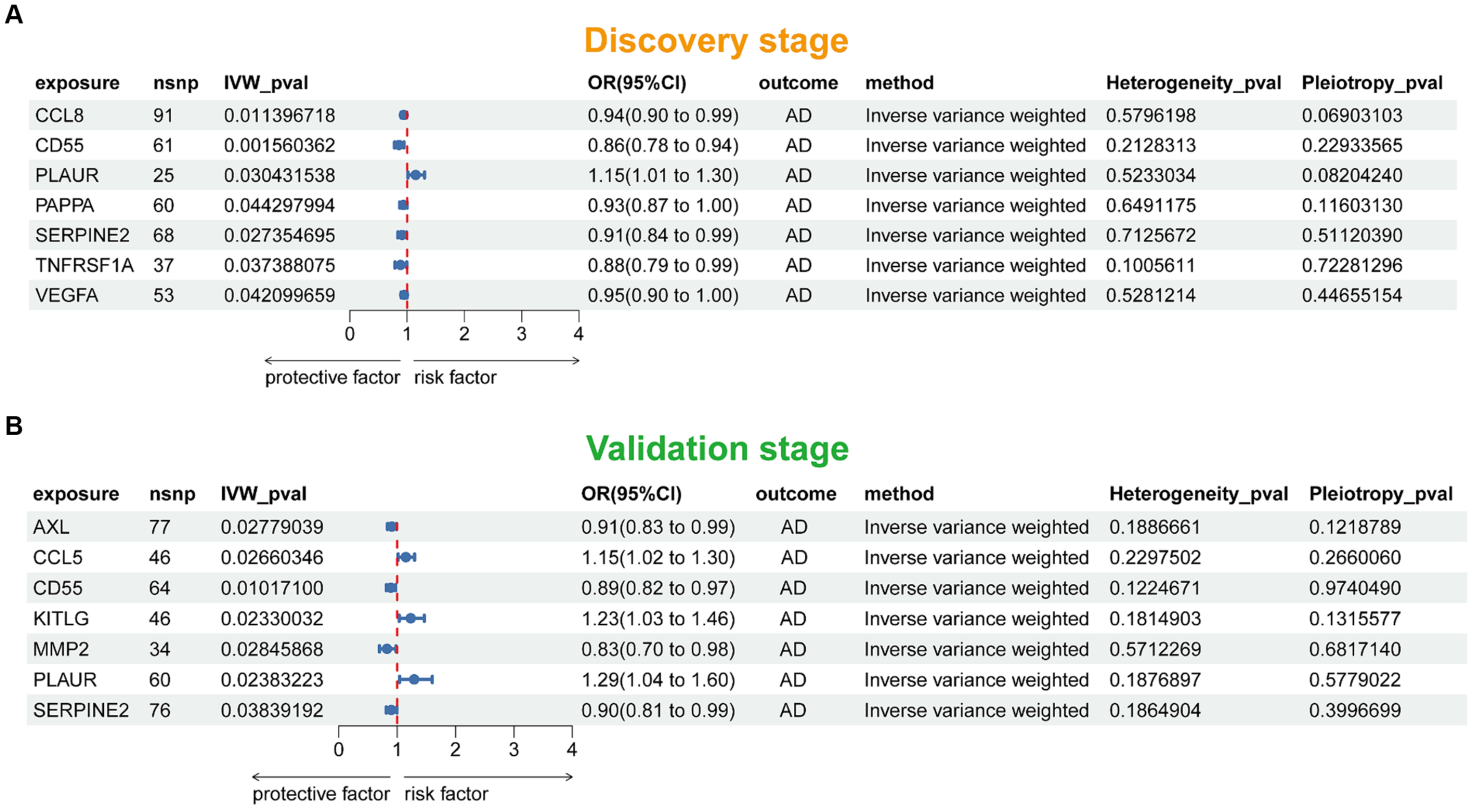
Forest plot of the MR results in discovery **(A)** and validation **(B)** stage. SNP, single nucleotide polymorphism; IVW, inverse-variance weighted; OR, odds ratio; AD, Alzheimer’s disease.

Figure 2 presented the flowchart of MR analysis. In the discovery stage, 7 SRPs were associated with risk of AD (Figure 6A; Supplementary Table 2), including: CCL8 (odds ratio [OR] = 0.94, 95% confidence interval [CI]: 0.90–0.99, IVW-*p* = 1.14E-2), CD55 (OR = 0.86, 95% CI: 0.78–0.94, IVW-*p* = 1.56E-3), PLAUR (OR = 1.15, 95% CI: 1.01–1.30, IVW-*p* = 3.04E-2), PAPPA (OR = 0.93, 95% CI: 0.87–1.00, IVW-*p* = 4.43E-2), SERPINE2 (OR = 0.91, 95% CI: 0.84–0.99, IVW-*p* = 2.74E-2), TNFRSF1A (OR = 0.88, 95% CI: 0.79–0.99, IVW-*p* = 3.74E-2), VEGFA (OR = 0.95, 95% CI: 0.90–1.00, IVW-*p* = 4.21E-2). The *p* values of MR-Egger intercept test and Cochran’s Q test are all beyond 0.05, indicating no heterogeneity or pleiotropy in these results.

### 3.3 Validation stage MR results

In this stage, 7 SRPs were associated with risk of AD (Figure 6B; Supplementary Table 3), including: AXL (OR = 0.91, 95% CI: 0.83–0.99, IVW-*p* = 2.78E-2), CCL5 (OR = 1.15, 95% CI: 1.02–1.30, IVW-*p* = 2.66E-2), CD55 (OR = 0.89, 95% CI: 0.82–0.97, IVW-*p* = 1.01E-2), KITLG (OR = 1.23, 95% CI: 1.03–1.46, IVW-*p* = 2.33E-2), MMP2 (OR = 0.83, 95% CI: 0.70–0.98, IVW-*p* = 2.85E-2); PLAUR (OR = 1.29, 95% CI: 1.04–1.60, IVW-*p* = 2.38E-2); SERPINE2 (OR = 0.90, 95% CI: 0.81–0.99, IVW-*p* = 3.84E-2). Also, the p values of MR-Egger intercept test and Cochran’s Q test are all beyond 0.05, indicating no heterogeneity or pleiotropy in these results. Notably, the effect of PLAUR, CD55, and SERPINE2 on the risk of AD was validated.

### 3.4 Potential drugs targeting PLAUR

We obtained approved and not approved drugs potentially interacting with PLAUR from DGIdb database, including LENOGRASTIM, PHORBOL 12-MYRISTATE 13-ACETATE, RUXOLITINIB RECOMBINANT HUMAN MONOCYTE CHEMOATTRACTANT PROTEIN-2, RECOMBINANT INTERFERON GAMMA, ADENOVIRUS VECTOR, and DIPHTHERIA TOXIN (Supplementary Table 4).

## 4 Discussion

AD is a neurodegenerative disease (NDD) predominantly affecting the elders, associated with cognitive impairment and decreased quality of life in affected individuals (Livingston et al., 2020). Identifying potential biomarker and drug targets for AD is important. Therefore, this study aimed to explore the association between SRGs and SRPs and the development of AD. As a results, MR analysis indicated that high plasma levels of 2 SRPs (CD55 and SERPINE2) were associated with decreased risk of AD. More importantly, *PLAUR* was found to be up-regulated in AD brain tissues and the high plasma level of PLAUR was associated with higher risk of AD, which may contribute to the development of preventive and treatment strategies for this disease.

In aging individuals, there is a decline in the homeostasis capacity, which contributes to the accumulation of harmful cellular stressors, such as oxidative stress and DNA damage and lead to aging-associated damage to cells (Yang et al., 2024). Cellular senescence has been recognized as a fundamental mechanism underlying aging in organisms. Aging-associated homeostatic alterations activates cellular senescence (Li et al., 2024) and triggers chronic inflammation if the senescent cells were not cleared in time and accumulated. Characteristics of senescent cells include profound chromatin, secretory phenotype changes, and increased expression of senescence markers (Tchkonia et al., 2013). Senescent cells can develop a senescence-associated secretory phenotype (SASP) (Coppé et al., 2008; Xu et al., 2015), which is associated with physical, metabolic, and cognitive decline (Boniewska-Bernacka et al., 2020; Diniz et al., 2021). In AD, the chronic inflammation caused by the accumulation of senescent cells induces synapse damage and contributes to cognitive decline (Saez-Atienzar and Masliah, 2020). Notably, the association between toxic Tau protein aggregation and cellular senescence has been reported (Musi et al., 2018). The accumulation of senescent cells could induce a harmful environment for neuronal cells, making them more susceptible to toxic protein aggregation, and induce proteinopathy, both contributing to neuron loss and cognitive decline in AD (Saez-Atienzar and Masliah, 2020). A previous study has showed that the formation of senescent cells precedes tau aggregation in the AD mouse model (Bussian et al., 2018). Cellular senescence, in combination with the toxic protein aggregation, contributes to a greater accumulation of harmful factor within the cells in the brain. *PLAUR*, firstly identified as an SRG by Amor et al. (2020) and further identified by Saul et al. (2022), was found to be up-regulated in AD brain and the plasma level of the protein encoded by this gene was associated with higher risk of AD, indicating the potential role of it in the development of AD.

*PLAUR* gene encodes a protein called uPAR (urokinase plasminogen activator receptor), which is involved in many biological processes (Ragno, 2006; Blasi and Sidenius, 2010). Notably, uPAR is involved in inflammation-associated pathways such as the migration of inflammatory cells to lesions in tissue. Besides the plasma membrane-binding form, uPAR has soluble forms generated and released by glycosyl-phosphatidyl-inositol (GPI)-directed phospholipase. The classical function of uPAR is binding its ligand uPA, leading to the activation of plasminogen and subsequent proteolysis. Besides its canonical roles, evidence has indicated the participation of uPAR in the development of brain diseases (Archinti et al., 2011). Changes in the expression of uPAR has been observed in multiple brain diseases (Bruneau and Szepetowski, 2011). For instance, expression analysis in epileptic conditions revealed increased expression of uPAR in hippocampal and frontal lobe neurons and other cell types including microglia and vascular endothelial cells (Iyer et al., 2010; Liu et al., 2010). The increased expression of uPAR in microglia has also been observed in various neurodegenerative conditions associated with inflammatory responses (Cunningham et al., 2009). In multiple sclerosis (MS), increased expression of uPAR on the inflammatory cells in the perivascular zones has been demonstrated (Gveric et al., 2001), which was considered to be associated with further infiltration of inflammatory cells into the lesion. Such an increase in uPAR expression in perivascular zone has also been reported in AD brain (Deininger et al., 2002). Additionally, Walker et al. reported the significantly increased uPAR protein levels in human brain tissues with AD compared with control cases (Walker et al., 2002). They further demonstrate that uPAR expression is up-regulated following incubation of microglia obtained from human post-mortem brain using Aβ peptide (Walker et al., 2002). These results provide a connection between microglial activation and the possible involvement of uPAR in the pathogenesis of AD. Collectively, the association between *PLAUR* and the development of AD observed in this study might be bridged by the pro-inflammatory effects (potentially associated with microglial activation) of it in the central nervous system (CNS). Notably, despite two SNPs located in the *PLAUR* gene were indicative of a trend toward association, Cetinsoy et al. (2024) reported no significant genetic association between polymorphisms across the *PLAUR* gene and AD by utilizing the DNA bank of the Brains for Dementia Research cohort. This conflicting result might be caused by the differences between the study design and participants and highlighted the importance of our findings to be confirmed by more larger and prospective studies.

CD55, also known as Complement decay-accelerating factor, belongs to complement regulatory proteins and can protect cells from systemic complement (Flückiger et al., 2018). The deficiency of CD55 can lead to the increased sensitivity of cells to complement-mediated destruction (Merrill and Brodsky, 2018; Gurnari et al., 2021). It has also been reported that CD55 can control the adverse immune responses and neuroinflammation in CNS (Hoarau et al., 2011; Hernangómez et al., 2014). Notably, previous study found that a somatic mutation influencing transcription factor binding upstream of *CD55* gene contributes to AD pathogenesis by affecting the complement system (Helgadottir et al., 2019). Considering the identified causal effect of plasma CD55 on lower AD risk in this study, the mechanism underlying this effect might be bridged by the modulation of complement system and neuroinflammation.

*SERPINE2* (Serpin Family E Member 2), also termed as Protease Nexin-1 (*PN-1*), is a member of the serpin family of proteins, which can inhibit serine proteases including thrombin, urokinase, plasmin, and trypsin (Madjene et al., 2021), whose role in AD has not been fully understood. It presents strong antithrombotic and antifibrinolytic properties (Madjene et al., 2021). It is demonstrated that *SERPINE2* is constitutively secreted through small vesicles and plays a key role for synaptic plasticity in the developing and adult CNS (Giau et al., 2005). It has also been reported that *SERPINE2* is highly expressed by many cell types, especially microglia, and loss of SERPINE2 can lead to behavioral changes as well as deficits in synaptic plasticity in CNS (Krawczyk et al., 2023). Studies have also noticed the neurotrophic properties of SERPINE2 (Winokur et al., 2017), which can promote neurite outgrowth and extension (Choi et al., 1995). In addition, SERPINE2 is associated with coagulation and cholesterol homeostasis, whose mutations show pleiotropic effects on blood-cell-related traits such as platelet count, metabolic traits such as levels of lipoprotein and lipid, and on disease traits such as coronary artery disease and type 2 diabetes (Nath et al., 2019). Collectively, the effects of SERPINE2 on neurotrophy, synaptic plasticity, and neurite extension, as well as its influences on coagulation and lipid metabolism, might be the mechanical basis of the association between plasma SERPINE2 and lower risk of AD.

There were some limitations in this MR-designed investigation. First, this study only included European-ancestry individuals, which suggests that our findings cannot be directly applied to other populations. Second, our findings only revealed the causality between plasma levels of several SRPs and AD, not the underlying mechanisms, which are required to be elucidated by further studies.

In conclusion, by combing transcriptomic and MR analysis, we provided the evidence that *PLAUR*, as an SRG, may play a role in the development of AD, which may provide the rationale of existing drugs or novel drugs for AD, such as LENOGRASTIM and RUXOLITINIB (Cannon et al., 2024). Still, the results require to be confirmed by further studies. Moreover, investigating the mechanism underlying the identified causality in our study would also help us understanding the pathogenesis of this disease.

## Data availability statement

The original contributions presented in the study are included in the article/Supplementary material, further inquiries can be directed to the corresponding author.

## Ethics statement

This study used only publicly available, deidentified summary statistics from previously published works, making it exempt according to the institutional review board (IRB) of Shengjing Hospital of China Medical University. Our research adhered to the tenets of the Declaration of Helsinki.

## Author contributions

YL: Writing – original draft, Visualization, Project administration, Methodology, Investigation, Formal analysis, Data curation, Conceptualization. JC: Writing – review & editing, Visualization, Supervision, Project administration, Methodology, Investigation, Formal analysis, Data curation, Conceptualization.

## Footnotes

^1^https://dgidb.org

## References

[ref1] Amor C. Feucht J. Leibold J. Ho Y. J. Zhu C. Alonso-Curbelo D.. (2020). Senolytic CAR T cells reverse senescence-associated pathologies. Nature 583, 127–132. doi: 10.1038/s41586-020-2403-9, PMID: 32555459 PMC7583560

[ref2] Archinti M. Britto M. Eden G. Furlan F. Murphy R. Degryse B. (2011). The urokinase receptor in the central nervous system. CNS Neurol. Disord. Drug Targets 10, 271–294. doi: 10.2174/187152711794480393 20874700

[ref3] Blasi F. Sidenius N. (2010). The urokinase receptor: focused cell surface proteolysis, cell adhesion and signaling. FEBS Lett. 584, 1923–1930. doi: 10.1016/j.febslet.2009.12.039, PMID: 20036661

[ref4] Boniewska-Bernacka E. Pańczyszyn A. Klinger M. (2020). Telomeres and telomerase in risk assessment of cardiovascular diseases. Exp. Cell Res. 397:112361. doi: 10.1016/j.yexcr.2020.112361, PMID: 33171154

[ref5] Bowden J. Del Greco M. F. Minelli C. Zhao Q. Lawlor D. A. Sheehan N. A.. (2019). Improving the accuracy of two-sample summary-data Mendelian randomization: moving beyond the NOME assumption. Int. J. Epidemiol. 48, 728–742. doi: 10.1093/ije/dyy258, PMID: 30561657 PMC6659376

[ref6] Brookmeyer R. Evans D. A. Hebert L. Langa K. M. Heeringa S. G. Plassman B. L.. (2011). National estimates of the prevalence of Alzheimer's disease in the United States. Alzheimers Dement. 7, 61–73. doi: 10.1016/j.jalz.2010.11.007, PMID: 21255744 PMC3052294

[ref7] Bruneau N. Szepetowski P. (2011). The role of the urokinase receptor in epilepsy, in disorders of language, cognition, communication and behavior, and in the central nervous system. Curr. Pharm. Des. 17, 1914–1923. doi: 10.2174/138161211796718198, PMID: 21711233

[ref8] Burgess S. Dudbridge F. Thompson S. G. (2016). Combining information on multiple instrumental variables in Mendelian randomization: comparison of allele score and summarized data methods. Stat. Med. 35, 1880–1906. doi: 10.1002/sim.6835, PMID: 26661904 PMC4832315

[ref9] Bussian T. J. Aziz A. Meyer C. F. Swenson B. L. van Deursen J. M. Baker D. J. (2018). Clearance of senescent glial cells prevents tau-dependent pathology and cognitive decline. Nature 562, 578–582. doi: 10.1038/s41586-018-0543-y, PMID: 30232451 PMC6206507

[ref10] Cannon M. Stevenson J. Stahl K. Basu R. Coffman A. Kiwala S.. (2024). DGIdb 5.0: rebuilding the drug-gene interaction database for precision medicine and drug discovery platforms. Nucleic Acids Res. 52, D1227–d1235. doi: 10.1093/nar/gkad1040, PMID: 37953380 PMC10767982

[ref11] Cetinsoy O. Anyanwu I. Krishnanand H. Natarajan G. Ramachandran N. Thomas A.. (2024). Gene Association study of the Urokinase plasminogen activator and its receptor gene in Alzheimer's disease. J. Alzheimers Dis. 99, 241–250. doi: 10.3233/JAD-231383, PMID: 38669542

[ref12] Choi B. H. Kim R. C. Vaughan P. J. Lau A. Van Nostrand W. E. Cotman C. W.. (1995). Decreases in protease nexins in Alzheimer's disease brain. Neurobiol. Aging 16, 557–562. doi: 10.1016/0197-4580(95)00060-R 8544905

[ref13] Coppé J. P. Patil C. K. Rodier F. Sun Y. Muñoz D. P. Goldstein J.. (2008). Senescence-associated secretory phenotypes reveal cell-nonautonomous functions of oncogenic RAS and the p53 tumor suppressor. PLoS Biol. 6, 2853–2868. doi: 10.1371/journal.pbio.0060301, PMID: 19053174 PMC2592359

[ref14] Cunningham O. Campion S. Perry V. H. Murray C. Sidenius N. Docagne F.. (2009). Microglia and the urokinase plasminogen activator receptor/uPA system in innate brain inflammation. Glia 57, 1802–1814. doi: 10.1002/glia.20892, PMID: 19459212 PMC2816357

[ref15] Davey Smith G. Hemani G. (2014). Mendelian randomization: genetic anchors for causal inference in epidemiological studies. Hum. Mol. Genet. 23, R89–R98. doi: 10.1093/hmg/ddu328, PMID: 25064373 PMC4170722

[ref16] Deininger M. H. Trautmann K. Magdolen V. Luther T. Schluesener H. J. Meyermann R. (2002). Cortical neurons of Creutzfeldt-Jakob disease patients express the urokinase-type plasminogen activator receptor. Neurosci. Lett. 324, 80–82. doi: 10.1016/S0304-3940(02)00168-4, PMID: 11983300

[ref17] Demakis G. J. (2007). Disability in Alzheimer's disease: causes, consequences, and economic considerations. J. Health Hum. Serv. Adm. 30, 292–305. doi: 10.1177/107937390703000302, PMID: 18236705

[ref18] Diniz B. S. Vieira E. M. Mendes-Silva A. P. Bowie C. R. Butters M. A. Fischer C. E.. (2021). Mild cognitive impairment and major depressive disorder are associated with molecular senescence abnormalities in older adults. Alzheimers Dement 7:e12129. doi: 10.1002/trc2.12129, PMID: 33816758 PMC8012242

[ref19] Doolittle M. L. Saul D. Kaur J. Rowsey J. L. Vos S. J. Pavelko K. D.. (2023). Multiparametric senescent cell phenotyping reveals targets of senolytic therapy in the aged murine skeleton. Nat. Commun. 14:4587. doi: 10.1038/s41467-023-40393-9, PMID: 37524694 PMC10390564

[ref20] Farr J. N. (2023). Skeletal senescence with aging and type 2 diabetes. Endocrinol. Metab. 38, 295–301. doi: 10.3803/EnM.2023.1727, PMID: 37312256 PMC10323162

[ref21] Ferkingstad E. Sulem P. Atlason B. A. Sveinbjornsson G. Magnusson M. I. Styrmisdottir E. L.. (2021). Large-scale integration of the plasma proteome with genetics and disease. Nat. Genet. 53, 1712–1721. doi: 10.1038/s41588-021-00978-w 34857953

[ref22] Flückiger R. Cocuzzi E. Nagaraj R. H. Shoham M. Kern T. S. Medof M. E. (2018). DAF in diabetic patients is subject to glycation/inactivation at its active site residues. Mol. Immunol. 93, 246–252. doi: 10.1016/j.molimm.2017.06.036, PMID: 28886871 PMC5884443

[ref23] Giau R. Carrette J. Bockaert J. Homburger V. (2005). Constitutive secretion of protease nexin-1 by glial cells and its regulation by G-protein-coupled receptors. J. Neurosci. 25, 8995–9004. doi: 10.1523/JNEUROSCI.2430-05.2005, PMID: 16192390 PMC6725596

[ref24] Gillespie P. O'Shea E. Cullinan J. Lacey L. Gallagher D. Ni Mhaolain A.. (2013). The effects of dependence and function on costs of care for Alzheimer's disease and mild cognitive impairment in Ireland. Int. J. Geriatr. Psychiatry 28, 256–264. doi: 10.1002/gps.3819 23386588

[ref25] Gurnari C. Nautiyal I. Pagliuca S. (2021). Current opinions on the clinical utility of Ravulizumab for the treatment of paroxysmal nocturnal hemoglobinuria. Ther. Clin. Risk Manag. 17, 1343–1351. doi: 10.2147/TCRM.S273360, PMID: 34934322 PMC8684432

[ref26] Gveric D. Hanemaaijer R. Newcombe J. van Lent N. Sier C. F. Cuzner M. L. (2001). Plasminogen activators in multiple sclerosis lesions: implications for the inflammatory response and axonal damage. Brain 124, 1978–1988. doi: 10.1093/brain/124.10.1978, PMID: 11571216

[ref27] Helgadottir H. T. Lundin P. Wallén Arzt E. Lindström A. K. Graff C. Eriksson M. (2019). Somatic mutation that affects transcription factor binding upstream of CD55 in the temporal cortex of a late-onset Alzheimer disease patient. Hum. Mol. Genet. 28, 2675–2685. doi: 10.1093/hmg/ddz085 31216356 PMC6688063

[ref28] Hemani G. Zheng J. Elsworth B. Wade K. H. Haberland V. Baird D.. (2018). The MR-base platform supports systematic causal inference across the human phenome. eLife 7:7. doi: 10.7554/eLife.34408 PMC5976434 29846171

[ref29] Hernangómez M. Carrillo-Salinas F. J. Mecha M. Correa F. Mestre L. Loría F.. (2014). Brain innate immunity in the regulation of neuroinflammation: therapeutic strategies by modulating CD200-CD200R interaction involve the cannabinoid system. Curr. Pharm. Des. 20, 4707–4722. doi: 10.2174/1381612820666140130202911, PMID: 24588829 PMC4157566

[ref30] Hoarau J. J. Krejbich-Trotot P. Jaffar-Bandjee M. C. das T. Thon-Hon G. V. Kumar S.. (2011). Activation and control of CNS innate immune responses in health and diseases: a balancing act finely tuned by neuroimmune regulators (NIReg). CNS Neurol. Disord. Drug Targets 10, 25–43. doi: 10.2174/187152711794488601, PMID: 21143144

[ref31] Holloway K. Neherin K. Dam K. U. Zhang H. (2023). Cellular senescence and neurodegeneration. Hum. Genet. 142, 1247–1262. doi: 10.1007/s00439-023-02565-x 37115318

[ref32] Iyer A. M. Zurolo E. Boer K. Baayen J. C. Giangaspero F. Arcella A.. (2010). Tissue plasminogen activator and urokinase plasminogen activator in human epileptogenic pathologies. Neuroscience 167, 929–945. doi: 10.1016/j.neuroscience.2010.02.047, PMID: 20219643

[ref33] Krawczyk M. C. Godoy M. Vander P. Zhang A. J. Zhang Y. (2023). Loss of serpin E2 alters antimicrobial gene expression by microglia but not astrocytes. Neurosci. Lett. 811:137354. doi: 10.1016/j.neulet.2023.137354 37348749 PMC11473033

[ref34] Kurki M. I. Karjalainen J. Palta P. Sipilä T. P. Kristiansson K. Donner K.. (2003). FinnGen: unique genetic insights from combining isolated population and national health register data. doi: 10.1101/2022.03.03.22271360 [Preprint].

[ref35] Lambert J. C. Ibrahim-Verbaas C. A. Harold D. Naj A. C. Sims R. Bellenguez C.. (2013). Meta-analysis of 74,046 individuals identifies 11 new susceptibility loci for Alzheimer's disease. Nat. Genet. 45, 1452–1458. doi: 10.1038/ng.2802, PMID: 24162737 PMC3896259

[ref36] Lawlor D. A. Harbord R. M. Sterne J. A. Timpson N. Davey Smith G. (2008). Mendelian randomization: using genes as instruments for making causal inferences in epidemiology. Stat. Med. 27, 1133–1163. doi: 10.1002/sim.3034, PMID: 17886233

[ref37] Lei S. Hu M. Wei Z. (2024). Identification of systemic biomarkers and potential drug targets for age-related macular degeneration. Front. Aging Neurosci. 16:1322519. doi: 10.3389/fnagi.2024.1322519 38361503 PMC10867226

[ref38] Li D. Yu Q. Wu R. Tuo Z. Wang J. Ye L.. (2024). Interactions between oxidative stress and senescence in cancer: mechanisms, therapeutic implications, and future perspectives. Redox Biol. 73:103208. doi: 10.1016/j.redox.2024.103208, PMID: 38851002 PMC11201350

[ref39] Li G. S. Yang Y. Z. Ma G. R. Li P. F. Cheng Q. H. Zhang A. R.. (2023). Rheumatoid arthritis is a protective factor against Alzheimer's disease: a bidirectional two-sample Mendelian randomization study. Inflammopharmacology 32, 863–871. doi: 10.1007/s10787-023-01397-5 38151584

[ref40] Liu B. Zhang B. Wang T. Liang Q. C. Jing X. R. Zheng J.. (2010). Increased expression of urokinase-type plasminogen activator receptor in the frontal cortex of patients with intractable frontal lobe epilepsy. J. Neurosci. Res. 88, 2747–2754. doi: 10.1002/jnr.22419, PMID: 20648659

[ref41] Livingston G. Huntley J. Sommerlad A. Ames D. Ballard C. Banerjee S.. (2020). Dementia prevention, intervention, and care: 2020 report of the lancet commission. Lancet 396, 413–446. doi: 10.1016/S0140-6736(20)30367-6, PMID: 32738937 PMC7392084

[ref42] Madjene C. Boutigny A. Bouton M. C. Arocas V. Richard B. (2021). Protease Nexin-1 in the cardiovascular system: wherefore art thou? Front. Cardiovasc. Med. 8:652852. doi: 10.3389/fcvm.2021.652852, PMID: 33869311 PMC8044347

[ref43] Merrill S. A. Brodsky R. A. (2018). Complement-driven anemia: more than just paroxysmal nocturnal hemoglobinuria. Hematology Am. Soc. Hematol. Educ. Program 2018, 371–376. doi: 10.1182/asheducation-2018.1.371, PMID: 30504334 PMC6245985

[ref44] Musi N. Valentine J. M. Sickora K. R. Baeuerle E. Thompson C. S. Shen Q.. (2018). Tau protein aggregation is associated with cellular senescence in the brain. Aging Cell 17:e12840. doi: 10.1111/acel.12840, PMID: 30126037 PMC6260915

[ref45] Nath A. P. Ritchie S. C. Grinberg N. F. Tang H. H. F. Huang Q. Q. Teo S. M.. (2019). Multivariate genome-wide association analysis of a cytokine network reveals variants with widespread immune, Haematological, and Cardiometabolic pleiotropy. Am. J. Hum. Genet. 105, 1076–1090. doi: 10.1016/j.ajhg.2019.10.001, PMID: 31679650 PMC6904835

[ref46] Newman A. M. Gallo N. B. Hancox L. S. Miller N. J. Radeke C. M. Maloney M. A.. (2012). Systems-level analysis of age-related macular degeneration reveals global biomarkers and phenotype-specific functional networks. Genome Med. 4:16. doi: 10.1186/gm315, PMID: 22364233 PMC3372225

[ref47] Ning P. Guo X. Qu Q. Li R. (2023). Exploring the association between air pollution and Parkinson's disease or Alzheimer's disease: a Mendelian randomization study. Environ. Sci. Pollut. Res. Int. 30, 123939–123947. doi: 10.1007/s11356-023-31047-w 37995032

[ref48] Ragno P. (2006). The urokinase receptor: a ligand or a receptor? Story of a sociable molecule. Cell. Mol. Life Sci. 63, 1028–1037. doi: 10.1007/s00018-005-5428-1, PMID: 16465446 PMC11136413

[ref49] Saez-Atienzar S. Masliah E. (2020). Cellular senescence and Alzheimer disease: the egg and the chicken scenario. Nat. Rev. Neurosci. 21, 433–444. doi: 10.1038/s41583-020-0325-z, PMID: 32601397 PMC12548380

[ref50] Saul D. Kosinsky R. L. Atkinson E. J. Doolittle M. L. Zhang X. LeBrasseur N. K.. (2022). A new gene set identifies senescent cells and predicts senescence-associated pathways across tissues. Nat. Commun. 13:4827. doi: 10.1038/s41467-022-32552-1, PMID: 35974106 PMC9381717

[ref51] Tchkonia T. Zhu Y. van Deursen J. Campisi J. Kirkland J. L. (2013). Cellular senescence and the senescent secretory phenotype: therapeutic opportunities. J. Clin. Invest. 123, 966–972. doi: 10.1172/JCI64098, PMID: 23454759 PMC3582125

[ref52] Verbanck M. Chen C. Y. Neale B. do R. (2018). Detection of widespread horizontal pleiotropy in causal relationships inferred from Mendelian randomization between complex traits and diseases. Nat. Genet. 50, 693–698. doi: 10.1038/s41588-018-0099-7, PMID: 29686387 PMC6083837

[ref53] Walker D. G. Lue L. F. Beach T. G. (2002). Increased expression of the urokinase plasminogen-activator receptor in amyloid beta peptide-treated human brain microglia and in AD brains. Brain Res. 926, 69–79. doi: 10.1016/S0006-8993(01)03298-X, PMID: 11814408

[ref54] Winokur P. N. Subramanian P. Bullock J. L. Arocas V. Becerra S. P. (2017). Comparison of two neurotrophic serpins reveals a small fragment with cell survival activity. Mol. Vis. 23, 372–384, PMID: 28706437 PMC5501690

[ref55] Xu M. Palmer A. K. Ding H. Weivoda M. M. Pirtskhalava T. White T. A.. (2015). Targeting senescent cells enhances adipogenesis and metabolic function in old age. eLife 4:e12997. doi: 10.7554/eLife.12997, PMID: 26687007 PMC4758946

[ref56] Yang J. Luo J. Tian X. Zhao Y. Li Y. Wu X. (2024). Progress in understanding oxidative stress, aging, and aging-related diseases. Antioxidants 13:394. doi: 10.3390/antiox13040394 38671842 PMC11047596

[ref57] Yu S. Chen M. Xu L. Mao E. Sun S. (2023). A senescence-based prognostic gene signature for colorectal cancer and identification of the role of SPP1-positive macrophages in tumor senescence. Front. Immunol. 14:1175490. doi: 10.3389/fimmu.2023.1175490, PMID: 37090726 PMC10115976

[ref58] Zhu H. Lu R. Zhou Q. du Z. Jiang Y. (2023). Relationship between sphingomyelin and risk of Alzheimer's disease: a bidirectional Mendelian randomization study. J. Alzheimers Dis. Rep. 7, 1289–1297. doi: 10.3233/ADR-230126 10.3233/ADR-230126 PMC10741972 38143776

